# Prevalence and characterization of *Salmonella* in two integrated broiler operations in Korea

**DOI:** 10.1186/s13620-018-0114-4

**Published:** 2018-01-15

**Authors:** Jong Su Ha, Kwang Won Seo, Yeong Bin Kim, Min Su Kang, Chang-Seon Song, Young Ju Lee

**Affiliations:** 1 Samhwa GPS Breeding Agri. Inc., 32291, Hongseong, South Korea; 2 Department of Public Health, College of Veterinary Medicine, Kyungpook National University, 41566, Daegu, South Korea; 3 Animal and Plant Quarantine Agency, Ministry of Agriculture, Food and Rural Affairs, 39660, Gimcheon, South Korea; 4 Avian Disease Laboratory, College of Veterinary Medicine, Konkuk University, 05029, Seoul, South Korea

**Keywords:** Salmonella, Prevalence, Integrated broiler operation, PFGE, Chicken

## Abstract

**Background:**

Vertical integration of the broiler industry allows producers to combine different biosecurity and sanitation practices, housing technologies, and feeding regimens to improve food safety. The purpose of this study was to investigate the prevalence and distribution of *Salmonella,* to determine the source of *Salmonella* contaminants, and to reveal the relationships between isolates at each step in the vertically integrated broiler production system in two representative integrated broiler companies in Korea.

**Results:**

A total of 2148 samples were collected from 2 broiler breeder hatcheries, 14 broiler breeder farms, 3 broiler hatcheries, 16 broiler farms, 8 broiler transporting trucks and 6 slaughterhouses belonging to representative integrated broiler companies, and 205 (9.5%) of these samples were positive for *Salmonella*. The *Salmonella* prevalence in broiler hatcheries (34.0%) and broiler transporting trucks (62.5%) was higher (*P* < 0.05) whereas that in the broiler breeder hatchery (0.8%) was lower (*P* < 0.05), than the overall prevalence. Nine and 13 different *Salmonell*a serotypes were isolated from integrated companies A and B, and the predominant serotypes were *S.* Virchow (39.7%) and *S.* Hadar (59.2%), respectively. Pulsed field gel electrophoresis patterns of isolates from the two operations showed significant genetic relatedness within a single system.

**Conclusions:**

In a comparison of the two operations that participated in this study, the prevalence of *Salmonella* differed significantly between the broiler breeder hatchery, and broiler hatcheries and broiler farms.

## Background

*Salmonella* (NTS) are recognized as zoonotic agents and one of the most important foodborne pathogens [23]. Other foodborne pathogens are also considered very important for poultry (e.g Campylobacter). Many foods, particularly poultry and foods originating from poultry, are important sources of food-borne illnesses in humans [13, 25]. *Salmonella* (23%) is the major cause of bacterial food-borne poisoning in Korea [18]. Cheong et al. [4] reported that poultry are one of the major reservoirs of human *Salmonella* and Hyeon et al. [10] also reported that chickens and their meat had the highest *Salmonella* isolation rates among all livestock in Korea.

Dissemination of *Salmonella* in poultry breeding pyramids have been described previously and is thought to have contributed to the original dissemination of *Salmonella* to commercial broiler flocks [24, 28]. In general, broiler industry can be divided into the primary breeding sector and the production sector. The production sector range from parent stock to the processing plant and the primary breeding sector from pedigree to grandparent stock. According to a report of the Korea Institute for Animal Products Quality Evaluation [14], a few large integrated companies in Korea own grandparent stock among the primary breeding sector as well as the production sector. In addition, most integrated companies are vertically integrated. *Salmonella* control in integrated broiler chicken operations is complicated, because there are numerous potential sources of *Salmonella* contaminants in an integrated poultry system, including chicks, feed, rodents, wild birds, insects, trucks, and the farm and processing plant environments [2].

All sources of *Salmonella* are potentially important, but it is critical to evaluate the relative importance of different sources in specific management and environmental conditions. The purpose of this study was to investigate the prevalence and distribution of *Salmonella,* to determine the source of *Salmonella* contaminants, and to reveal the relationships between isolates at each step in a vertically integrated broiler production system based on two representative integrated broiler companies in Korea.

## Methods

### Sample collection

A total of 254, 1022, 206, 602, 8 and 128 samples were collected from 2 broiler breeder hatcheries, 14 broiler breeder farms, 3 broiler hatcheries, 16 broiler farms, 8 broiler transport trucks, and 6 slaughterhouses, respectively. All hatcheries, farms, trucks, and slaughterhouses belonged to two representative integrated broiler companies in Korea and were sampled during 10 to 30 visits in each location from 2010 to 2014. Samples were collected from hatcheries by one drag sampling according to a method described by Bailey et al. [2]. Briefly, approximately 300 cm^2^ in the designated hatcher area was drag swabbed, and each swab placed in a plastic bag. Samples from farms were obtained from dust and feces in accordance with the standard method of the National Poultry Improvement Plan [19]. Briefly, 15 different areas were swabbed per flock with 10 g of dust collected for each sample. Approximately 10 g of feces were also sampled from 15 different locations. Samples from crates and the outside surfaces of transport trucks were collected using the swab method after broiler chickens were unloaded. The rinse water from carcasses in the slaughterhouse were collected according to guidelines of the United States Department of Agriculture, Food Safety and Inspection Service [1]. Four carcasses were sampled from each farm, and each sample was aseptically transferred to a vacuum bag (Cryovag; Sealed Air, USA), 400 ml of sterile buffered peptone water (BPW; Difco, USA) was added, and the bag was shaken 50 times, and approximately 50 ml of rinse water was poured into a sterile specimen cup.

### Isolation and identification of *Salmonella*

Swab samples, feces and 25 ml of rinse water were added to 225 ml of BPW and incubated at 35 ± 2 °C for 20–24 h. After pre-enrichment of the BPW, 0.1 ml of the broth was transferred to a 10 ml of Rappaport-Vassiliadis broth (RV broth; Difco), that was prepared according to the manufacturer’s instructions. The RV broth was incubated overnight at 41.5 °C and streaked onto Rambach agar (Difco). Three typical colonies picked from a plate were serotyped by slide and tube agglutination methods using O and H antisera (Difco) according to the Kauffmann and White scheme [20]. If three colonies from same plate indicated the same serotype, one colony was randomly chosen for inclusion in this study.

### Pulsed field gel electrophoresis (PFGE)

PFGE was performed according to the “One-Day (24–28 hr) Standardized Laboratory Protocol for Molecular Subtyping of Non-typhoidal *Salmonella* by PFGE” [21]. A single colony of each isolate was streaked on tryptic soy agar (TSA) and incubated overnight at 37 °C. Using a cotton swab, a portion of the growth on agar plate was transferred to 2 ml of Cell Suspension Buffer (100 mM Tris: 100 mM EDTA, pH 8.0) and the concentration of cell suspensions adjusted to 14–15% in a bioMerieux Vitek colorimeter. Immediately, 400 μl of adjusted cell suspension was transferred to 1.5 ml micro-centrifuge tubes with 20 μl of proteinase K (20 mg/ml stock), subsequently mixed with 400 μl of melted 1% SeaKem Gold (Cambrex, East Rutherford, NJ): 1% SDS agarose was prepared with TE Buffer (10 mM Tris: 1 mM EDTA, pH 8.0), and pipetted into disposable plug moulds. Three plugs were transferred to 50 ml polypropylene screwtubes with 5 ml of Cell Lysis Buffer (50 mM Tris: 50 mM EDTA, pH 8.0 with sarcosyl) and 25 μl of proteinase K (20 mg/ml stock) and incubated at 54 °C in a shaker water bath for 2 h with agitation. Thereafter, the plugs were washed twice with 15 ml of sterile water and three more times with TE Buffer at 50 °C for 15 min. Chromosomal DNA was digested with 50 U of *Xba*I (Promega, Madison, WI, USA.), and PFGE was performed on a CHEF Mapper XA System (Bio-Rad Lab., Richmond, CA, USA) in 0.5X Tris-Borate-EDTA buffer (Bio-Rad Lab.) with water circulation at 14 °C. Pulse times were ramped from 2.2 to 63.8 s during an 18 h run at 6.0 V/cm. After electrophoresis, the gels were stained within 2 μg of aqueous ethidium bromide (Sigma-Aldrich. St. Louis, MO, USA) per ml for 15 min and were photographed using 300 nm UV light. Similarities in PFGE patterns were calculated by using a computer-based similarity and clustering program (BioNumerics 3.0, Applied Maths, Biosistematica, Devon, UK). The dice coefficient was used to express similarities, and a similarity matrix was shown graphically by using the unweighted pair group method with arithmetic mean (UPGMA). The relatedness of the PFGE profiles of *Salmonella* isolates was estimated based on the presence or absence of shared bands. A PFGE type was defined as a group of isolates with similarities ≥85% [12].

## Results

Table 1 shows the *Salmonella* isolates obtained from two representative integrated broiler companies in Korea. A total of 2148 samples were collected from 2 broiler breeder hatcheries, 14 broiler breeder farms, 3 broiler hatcheries, 16 broiler farms, 8 broiler transporting trucks and 6 slaughterhouses belonging to representative integrated broiler companies, 205 (9.5%) of these samples were positive for *Salmonella*. The *Salmonella* prevalence in the broiler hatchery (34.0%) and broiler transporting truck (62.5%) was higher (*P* < 0.05) whereas that in the broiler breeder hatchery (0.8%) was lower (*P* < 0.05), than the overall prevalence. In integrated broiler company A, *Salmonella* were most frequently found in the broiler farms (33.3%) and broiler transporting trucks (33.3%), followed by the broiler hatcheries (22.2%), broiler breeder farms (19.0%), carcasses (13.3%), and broiler breeder hatchery (1.6%). In integrated broiler company B, *Salmonella* were most frequently found in broiler transporting trucks (80.0%), followed by the broiler hatcheries (44.9%), broiler breeder farms (14.7%), broiler farms (13.3%) and carcasses (13.3%), and *Salmonella* was not isolated from the broiler breeder hatchery. In a comparison of the two companies, the *Salmonella* prevalence was significantly different between the broiler breeder hatchery, and broiler hatchery and broiler farm (*P* < 0.05).

**Table 1:** Prevalence of *Salmonella* spp. isolated from two representative integrated broiler operations

Sampling sites	No. (%) of sample contaminated *Salmonella*/No. of samples tested		
	Integrated broiler operation A	Integrated broiler operation B	Total
Broiler breeder hatchery	2/126 (1.6)^a*^	0/128 (0.0)^b*^	2/254 (0.8)^*^
Broiler breeder farm	16/84 (19.0)	138/938 (14.7)	154/1022 (15.1)
Broiler hatchery	22/99 (22.2)^b^	48/107 (44.9)^a*^	70/206 (34.0)^*^
Broiler farm	20/60 (33.3)^a*^	72/542 (13.3)^b^	92/602 (15.3)
Broiler transporting truck	1/3 (33.3)	4/5 (80.0)^*^	5/8 0 (62.5)^*^
Chicken slaughterhouses	5/36 (13.3)	12/92 (13.0)	17/128 (13.3)
Total	66/408 (16.2)	274/1812 (15.1)	205/2148 (9.5)

^ab^Prevalence values in the same row followed by different letters are different by the Fisher’s exact test (*P* < 0.05)

^*^P < 0.05 by Fisher’s exact test; as compared with total positive rate

Table 2 shows the distribution of *Salmonella* serotypes recovered from various stages in the two integrated broiler systems. Nine different *Salmonell*a serotypes were isolated from integrated company A, including *S.* Virchow (39.7%), *S*. Heidelberg (23.1%), *S.* Enteritidis (10.3%) and *S*. Hato (9.0%). Thirteen *Salmonella* serotypes were isolated from integrated company B, with *S.* Hadar (59.2%), *S.* Montevideo (14.5%) and *S.* Senftenberg (12.8%) the most prevalent types. The distribution of predominant serotypes differed between companies A and B.

**Table 2:** Distribution of *Salmonella* serotypes recovered from representing stages in the two integrated broiler operations (A and B)

Serotypes	No. (%) of *Salmonella* included													
	Broiler breeder hatchery		Broiler breeder farm		Broiler hatchery		Broiler farm		Broiler transporting truck		Chicken slaughterhouses		Total	
	A (*n* = 2)	B (*n* = 0)	A (*n* = 16)	B (*n* = 138)	A (*n* = 23)	B (*n* = 75)	A (*n* = 20)	B (*n* = 72)	A (n = 1)	B (*n* = 4)	A (n = 10)	B (*n* = 14)	A (*n* = 78)	B (*n* = 304)
Bradford			2 (12.5)										2 (2.6)	
Enteritidis					5 (21.7)	1 (1.3)	2 (10.0)			1 (25.0)	1 (10.0)	4 (28.6)	8 (10.3)	6 (2.0)
Hadar				133 (96.4)				43 (59.7)		1 (25.0)		3 (21.4)		180 (59.2)
Hato	2 (100)		1 (6.3)		2 (8.7)						2 (20.0)		7 (9.0)	
Havana				1 (0.7)										1 (0.3)
Heidelberg			12 (75.0)		5 (21.7)						1 (10.0)		18 (23.1)	
London								1 (1.4)						1 (0.3)
Menston						2 (2.7)								2 (0.7)
Montevideo						41 (51.4)					3 (30.0)	3 (21.4)	3 (3.8)	44 (14.5)
Newport												2 (14.3)		2 (0.7)
Reading									1 (100)	1 (25.0)			1 (1.3)	1 (0.3)
Senftenberg					1 (4.3)	31 (41.3)		6 (8.3)			2 (20.0)	2 (14.3)	3 (3.8)	39 (12.8)
Thompson								4 (5.6)						4 (1.3)
Typhimurium				2 (1.4)										2 (0.7)
Ughelli			1 (6.3)										1 (1.3)	
Vellore										1 (25.0)				1 (0.3)
Virchow					16 (69.6)		15 (75.0)	2 (2.8)					31 (39.7)	2 (0.7)
Untypable				2 (1.4)			3 (15.0)	16 (22.2)			1 (10.0)		4 (5.1)	19 (6.3)

*n* = No. of *Salmonella* isolated in this study

**Fig. 1:**
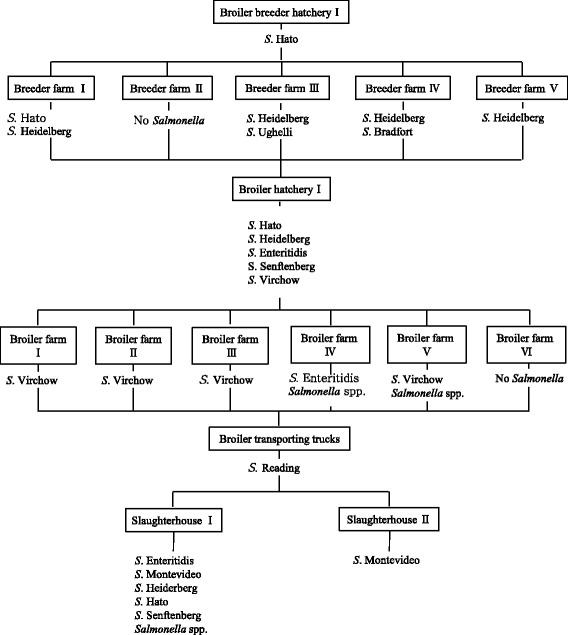
Transmission of *Salmonella* in the integrated broiler operation A

Figure 1 shows the distribution of the nine different serotypes of *Salmonella* isolates in integrated company A. *S*. Hato was found in the broiler breeder hatchery, broiler breeder farm, broiler hatchery, and chicken slaughterhouse, but was not found in the broiler farms and on the broiler transporting trucks. *S*. Heidelberg was isolated from 4 out of 5 broiler breeder farms, a broiler hatchery, and a chicken slaughterhouse, and *S*. Enteritidis was found in a broiler hatchery, 1 out of 6 broiler farms, and a chicken slaughterhouse. *S*. Virchow was predominant in broiler farms, but was not found in chicken slaughterhouses.

**Fig. 2:**
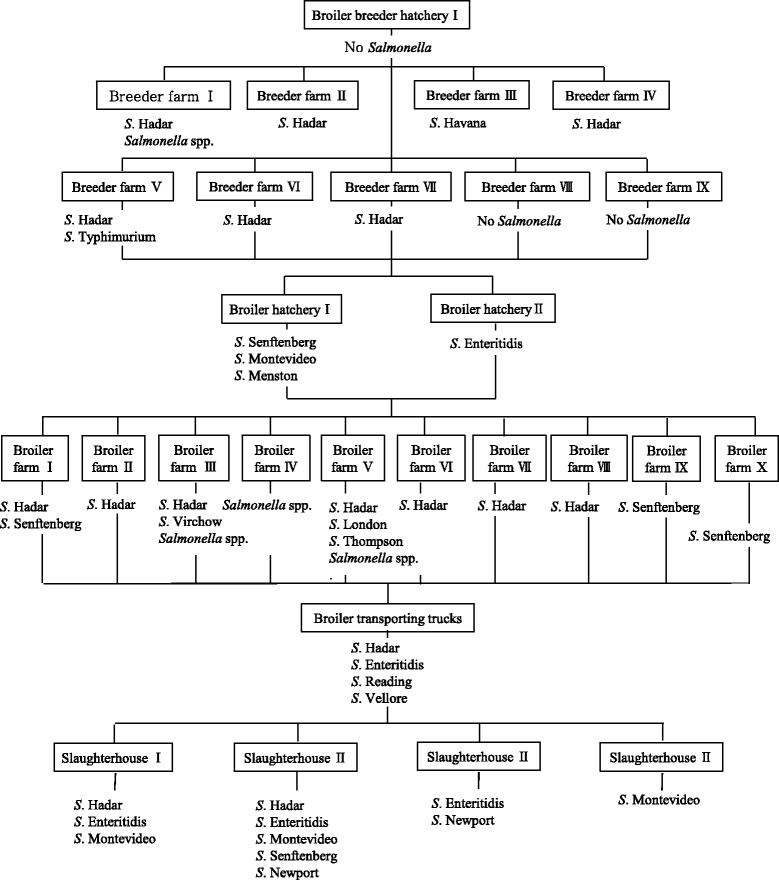
Transmission of *Salmonella* in the integrated broiler operation B

Figure 2 shows the distribution of the 13 different serotypes of *Salmonella* isolates in integrated company B. Although *Salmonella* was not found in the broiler breeder hatchery, *Salmonella* was observed in all subsequent stages of the broiler chicken production and processing system. *S*. Hadar was isolated from all phases of broiler chicken production and processing including the broiler breeder farms to chicken slaughterhouses. *S*. Enteritidis was found in 1 out of 2 broiler hatcheries, on the broiler transporting truck and in 3 out of 4 chicken slaughterhouses, but was not found in the 10 broiler farms. *S*. Typhimurium was only isolated from 1 out of 9 broiler breeder farms.

**Fig. 3:**
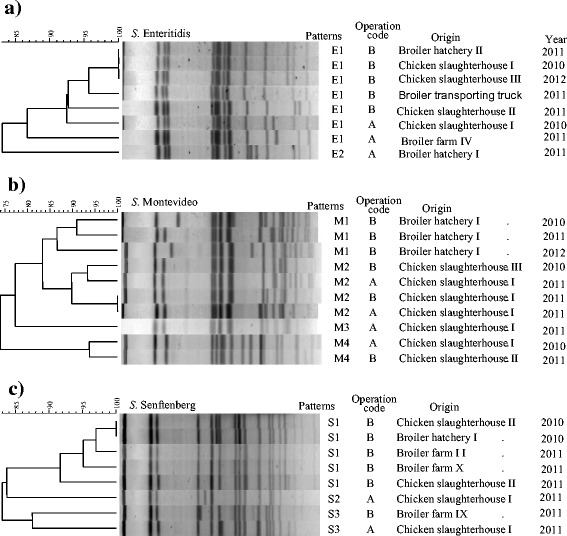
Dendrograms for *Salmonella* isolates by the restriction fragments created by *Xba*I enzyme. **a** *S.* Enteritidis, **b** *S*. Montevideo, **c** *S*. Senftenberg. The patterns were compared by UPGMA (unweighted pair group method with arithmetic mean) and defined as a group of isolates with a similarity of ≥85%

*S*. Enteritidis (*n* = 8), *S*. Montevideo (*n* = 10) and *S*. Senftenberg (n = 8), which were isolated frequently in company A and B, were further characterized by PFGE (Fig. 3). PFGE patterns of isolates from the two systems showed significant genetic relatedness within the same system. *S.* Enteritidis isolated from the hatchery and slaughterhouses of company B clusted together, even when they were isolated one different dates. *S.* Montevideo showed four PFGE patterns, with isolates from the same origin clustering together.

## Discussion

Although *Salmonella* infection in elite and grandparent chicken breeding flocks is extremely rare and is not considered a source of infection for the industry as a whole, Williams et al. [29] reported that contaminated eggs have the potential spread *Salmonella* through a hatchery, because eggs from infected flocks can be contaminated during laying, or from infected litter, dust, or equipment at the production site, and subsequently, *Salmonella* can disseminate throughout the integrated poultry industry. The prevalence of *Salmonella* (0.8%) in broiler breeder hatcheries in this study was relatively low, but even infected breeder flock can cause widespread *Salmonella* contamination [27]. Furthermore, the prevalence of *Salmonella* (15.1%) in broiler breeder farms observed in this study was relatively high compared to that in broiler breeder hatcheries.

The presence and persistence of *Salmonella* contaminants in commercial hatcheries indicate that the vulnerable day-of-hatch chick may be at greater risk of colonization in the hatchery than during grow-out [6]. Broiler farms usually have a higher prevalence of microorganisms, than other components of the production system because of the relatively poor sanitation management practices on these farms [11]. The prevalence of *Salmonella* in broiler farms in this study was 15.3%. In other countries, the prevalence of *Salmonella* in broiler farms range from 9.8% [2] and 13.5% [16] in the United States, to 4.7% and 7.2% in the Netherlands [26], to less than 1% in Finland and Sweden [8], and to as much as 68.2% in Hungary [8].

Previous studies have shown that transport vehicles and crates may play a critical role in transferring *Salmonella* between broiler farms and slaughterhouses, and that they are important sources of *Salmonella* contamination for batches of birds and farms [5, 17] because of exposure to the contaminated environments of broiler farms and slaughterhouses. Hald et al. [9] reported that the level of contamination dramatically increases during containment in holding cages before slaughter. In this study, broiler transporting trucks had the highest prevalence of *Salmonella* (62.5%) of all phases in the broiler supply chain.

In this study, 13.3% of carcasses from chicken slaughterhouses were positive for *Salmonella*. Lee et al. [15] reported that the prevalence of *Salmonella* in whole chicken carcasses from slaughterhouses was 15.5%, which was lower than the prevalence (42.7~58.3%) reported in studies conducted before 2011 in Korea [1, 30].

In a comparison of the two operations that participated in this study, the prevalence of *Salmonella* differed significantly between the broiler breeder hatchery, and broiler hatcheries and broiler farms. *Salmonella* prevalence might be associated with differences in hygiene and sanitation levels. Vertical integration of the broiler industry allows producers to combine different biosecurity and sanitation practices, housing technologies and feeding regimens to improve food safety. This structure allows a greater ability to govern each aspect of food safety from the breeder farm to the hatchery to the processing plant. Therefore, vertical integration has allowed strict maintenance of biosecurity measures, vaccination programs, and testing for bacteria such as *Salmonella* at breeder farms and hatcheries.

The predominant serotypes differed between the two operations surveyed. The distribution of *Salmonella* serotypes may have been affected by inter- and intra-regional differences [2, 3, 7, 22]. The two systems compared in this study were located in the northwest and central areas of Korea. Circulation of *Salmonella* in these systems may also have been related to farm density, little downtime, and concurrent disease, along with regional density and, poor sanitation and management practices.

PFGE is a useful way to trace *Salmonella* dissemination within a system. PFGE patterns of *S*. Enteritidis, *S*. Montevideo and *S*. Senftenberg isolated in this study showed significant genetic relatedness within the same system. A number of isolates from farms, hatcheries, and slaughterhouses had PFGE patterns in common, suggesting that many *Salmonella* strains are transmitted back through stage in the production process. Highly consistent PFGE patterns from different sources and dates within same operation suggest that the propagation of *Salmonella* clones through the broiler supply chain could spread antibiotic resistance and virulence genes.

## Conclusions

In conclusion, this study provides a prevalence and characterization of *Salmonella* of all phases in the broiler supply chain over the past 5 years in Korea. In a comparison of the two operations that participated in this study, the prevalence of *Salmonella* differed significantly between the broiler breeder hatchery, and broiler hatcheries and broiler farms. Therefore, these data indicate the critical need to control *Salmonella* in breeder farms and hatcheries, and suggest important points for control of infection in large-scale poultry operations in Korea.

## Abbreviations

NTS: Non-typhoid *Salmonella*
PFGE: Pulsed field gel electrophoresis
*S*. Bradford: *Salmonella* Bradford
*S.* Enteritidis: *Salmonella* Enteritidis
*S*. Hadar: *Salmonella* Hadar
*S*. Hato: *Salmonella* Hato
*S*. Havana: *Salmonella* Havana
*S*. Heidelberg: *Salmonella* Heidelberg
*S*. London: *Salmonella* London
*S*. Menston: *Salmonella* Menston
*S.* Montevideo: *Salmonella* Montevideo
*S*. Newport: *Salmonella* Newport
*S*. Reading: *Salmonella* Reading
*S.* Senftenberg: *Salmonella* Senftenberg
*S*. Thompson: *Salmonella* Thompson
*S*. Typhimurium: *Salmonella* Typhimurium
*S*. Ughelli: *Salmonella* Ughelli
*S*. Vellore: *Salmonella* Vellore
*S*. Virchow: *Salmonella* Virchow
*Salmonella* spp.: *Salmonella* species
UPGMA: Unweighted pair group method with arithmetic mean
